# The association of parity/live birth number with incident type 2 diabetes among women: over 15 years of follow-up in The Tehran Lipid and Glucose Study

**DOI:** 10.1186/s12905-021-01519-7

**Published:** 2021-10-29

**Authors:** Seyyed Saeed Moazzeni, Reyhane Hizomi Arani, Samaneh Asgari, Fereidoun Azizi, Farzad Hadaegh

**Affiliations:** 1 Prevention of Metabolic Disorders Research Center, Research Institute for Endocrine Sciences, Shahid Beheshti University of Medical Sciences, Tehran, Iran; 2 Endocrine Research Center, Research Institute for Endocrine Sciences, Shahid Beheshti University of Medical Sciences, Tehran, Iran

**Keywords:** Live birth, Parity, Diabetes mellitus, Type 2

## Abstract

**Background:**

Childbearing may increase the future risk of developing type 2 diabetes mellitus (T2DM) in mothers. However, the issue is not clear completely and not investigated in the Middle East, a region with a high burden of T2DM. In the current study, we examined the association of parity/live birth number with incident T2DM among Iranian women.

**Methods:**

The study population included 2552 women aged 30–65 years recruited in 1999–2001 and were followed for incident T2DM by 3-year intervals. Multivariable Cox proportional hazard models were applied to estimate hazard ratios (HRs) and 95% confidence intervals (CIs) of the parity/live birth number for incident T2DM. Parity number was defined as the number of live childbirth (number of live birth) plus the number of stillbirth (defined as birth of an infant that died after the 20th week of pregnancy in the uterus).

**Results:**

During a median follow-up of 15.4 years, 557 incident T2DM cases have occurred. After adjustment for potential T2DM risk factors and reproductive factors, each additional parity caused a 9% higher risk for incident T2DM. Moreover, compared to women with one parity, those with 3 and ≥ 4 parity had HRs of 1.73 [95% CI: 1.06–2.83] and 2.23 [1.36–3.65], respectively. After further adjustment for body mass index (BMI) and waist circumference, although the HRs were attenuated prominently, parity ≥ 4 was associated with significantly higher risk (HR: 1.72 [1.05–2.83]); even after further adjustment for triglycerides (TG)/ high-density lipoprotein cholesterol (HDL-C), the risk remained marginally significant (HR: 1.64 [1.00–2.70; *P* value: 0.051]). For the number of live birth, the results were also similar. Moreover, in a sensitivity analysis, when we considered BMI change during follow-up as another covariate, generally, the effect sizes did not change; the trend of HRs across categories of parity number remained marginally significant (*P* value: 0.064).

**Conclusions:**

During a long-term follow-up, after adjustment for potential T2DM risk factors, reproductive factors, obesity indices, and TG/HDL-C (insulin resistance surrogate), we demonstrated that higher parity/live birth numbers could be associated with increased risk of T2DM development among Iranian women. Moreover, even after further adjustment for BMI change, the suggestive higher risk was still found.

**Supplementary Information:**

The online version contains supplementary material available at 10.1186/s12905-021-01519-7.

## Introduction

Diabetes mellitus (DM) is a leading cause of mortality and morbidity in the world, with a greater burden in low- and middle-income countries [1]. In 2017, the global prevalence number of DM reached 476.0 million, with a 129.7% increase from 1990 [1]. In addition to prevalence, the trends of incidence rate, death rate, and disability-adjusted life-years (DALYs) were also increasing for DM, especially for type 2 DM (T2DM) [1]. The age-standardized prevalence of T2DM among adult residents of Tehran was 13.5% in 2008–2011 [2], and about 1% of them developed T2DM annually [3].

In addition to known DM risk factors such as obesity, genetic susceptibility, unhealthy lifestyle, and environmental risk factors [4], it was suggested that childbearing could associate with T2DM development among mothers [5]. Pregnancy, as one of the most important events during women's life, involves marked alterations in cardiometabolic parameters, including weight gain, increased plasma glucose, insulin resistance, and dyslipidemia. Although these changes are physiological, which are beneficial to both mother and fetus, they may have an impact on the risk of diabetes and other cardiometabolic diseases in future life [5–8]. In a meta-analysis of prospective studies on this issue, the risk of T2DM was increased by 9% for each birth [5]. Similarly, in another meta-analysis, each additional birth increased the risk by 6% [9]. Moreover, in both meta-analyses, compared to those with lower parity, higher parity (at least 4 live births) was associated with about 40% higher risk for incident T2DM [5, 9]. It should be noted that in both of these meta-analyses, the authors found significant heterogeneities (all I^2^ > 70%) among previous cohort studies conducted in Europe, East Asia, and the US [5, 9]. The authors recommended further investigations with further adjustment for the potential confounders, especially among uninvestigated populations [5, 9], considering that the impact of parity number on incident T2DM is not same in different ethnicity [10]. To the best of our knowledge, there is a lack of information on this issue in the Middle East and North Africa (MENA) region, with a high burden of DM [11]. The current study aims at determining whether the parity/live birth number is an independent risk factor for T2DM development among Iranian women aged 30–65 years. We used collected data from the oldest cohort of the MENA region, the Tehran Lipid and Glucose Study (TLGS).

## Materials and methods

### Study design and study population

The TLGS is an ongoing cohort study carried out in a general population that resided in district 13 of Tehran. This study was initially designed to assess the prevalence and incidence of non-communicable diseases (NCDs) and their risk factors. Prevention of NCDs by advancing healthier lifestyles is another aim of the TLGS. For the current study, phase I of the TLGS (January 31, 1999- July 03, 2001) was considered as recruitment. Data gathering for follow-up was done up to phase VI in about 3-year intervals (i.e., phase II: 2001–2005, phase III: 2005–2008, phase IV: 2008–2011, phase V: 2011–2014, and phase VI: 2015–2018). Details of the design, methods, and enrollment strategy of the TLGS have been described elsewhere [12].

A total of 4023 women aged 30–65 years were initially recruited. Firstly, we excluded 547 participants with prevalent DM at baseline. Then 152 singles (never married) and 11 individuals with no live birth (n = 11) were also excluded from the analyses, leading to a total of 3313 women. Moreover, due to the lack of baseline data on fasting plasma glucose (FPG) and 2-h post-challenge plasma glucose (2h-PCPG), the glycemic status of 68 participants was unknown for us, and they were excluded. Other reasons for exclusion were missing data on parity/live birth number and other covariates (n = 202) and no follow-up measurement (n = 491). Finally, 2552 female participants (divorced or married) were eligible for our analysis (response rate: 77%).

### Clinical and laboratory measurements

Demographic data, past medical and drug history, family history of DM, smoking habits, education level, marital status, and data on the history of preeclampsia, macrosomia, and parity/live birth number were obtained by structured, interviewer-administered questionnaires at the enrollment phase (Additional file 1: Questionnaire). Levels of physical activity were determined by the Lipid Research Clinic (LRC) questionnaire [12]. We measured weight, height, waist circumference (WC), and blood pressure by standardized procedures according to the TLGS protocol [12]. Body mass index (BMI) was considered as weight in kilograms divided by height in meters squared. We determined systolic blood pressure (SBP) and diastolic blood pressure (DBP) as the mean of the two standardized measurements on the right arm by a sphygmomanometer after 15 min of rest. For laboratory measurements of each phase, we asked our participants to fast for at least 12 h before morning blood sample collection. Samples were analyzed on the same day in the TLGS research laboratory. For the 2h-PCPG test, 82.5 g glucose monohydrate solution (equivalent to 75 g anhydrous glucose) was orally taken by participants without a history of using glucose-lowering medications. 2h-PCPG, FPG, high-density lipoprotein cholesterol (HDL-C), and triglycerides (TG) were measured by validated and standard methods as previously explained [12].

### Definition of terms

We considered incident T2DM as the presence of at least one of the following criteria: (a) FPG level of ≥ 7.0 mmol/L, (b) 2 h-PCPG level of ≥ 11.1 mmol/L, (c) pharmacologically treated with glucose-lowering medications because of DM [13]. In our study, smoking status was categorized into never/former smokers versus current smokers. Participants were categorized based on their education levels into more than 12 years of education and less than 12 years of education. Having physical activity in less than 3 days of each week was considered as low physical activity [12]. Macrosomia was defined as a birth weight > 4 kg [14]. Parity number was defined as the number of live childbirth (number of live birth) plus the number of stillbirth (defined as birth of an infant that died after the 20th week of pregnancy in the uterus). History of miscarriage was positive if an embryo or fetus was lost before the 20th week of pregnancy.

### Statistical analyses

Baseline characteristics across the number of parity (1, 2, 3, and ≥ 4) are presented as mean ± standard deviation (SD) for normally distributed continuous variables, median (interquartile range: IQR) for the highly skewed variables, and number (%) for categorical variables. Comparing baseline characteristics among different groups was made using chi-square, fisher's exact, ANOVA, and Kruskal–Wallis tests as appropriate.

The time to event was defined as the time of censoring or the event occurring, whichever firstly came. We censored participants in the case of death, leaving the district, or being in the study until the end of phase VI (April 2018) without any event. For the censored individuals, the survival time was the interval between the first and the last observation dates. For the cases of incident T2DM, the event date was defined as the mid-time between the date of follow-up visit at which T2DM was detected for the first time, and the most recent follow-up visit preceding the diagnosis.

The hazard ratios (HRs) with 95% CIs are reported using the Cox proportional hazard models to evaluate the association of the parity/live birth number with incident T2DM in 5 models: Model 1: adjusted for age; Model 2: adjusted for age, educational level, low physical activity, family history of diabetes, SBP, DBP, and anti-hypertensive medications usage; Model 3: Model 2 + further adjusted for reproductive factors (history of macrosomia, preeclampsia, and oral contraceptive pill (OCP) usage); Model 4: Model 3 + further adjusted for BMI and WC; Model 5: Model 4 + further adjusted for TG/HDL-C. Confounders were well-known risk factors for incident T2DM [15], which were previously investigated in the TLGS [3]. Moreover, the *P* value for the trend was calculated by considering each parity and live birth category as a continuous variable.

We assessed the Cox models’ proportionality with the Schoenfeld residual test. All proportionality assumptions were appropriate, generally. Statistical analyses were done using STATA version 14 (StataCorp LP, College Station, Texas) statistical software. *P* values < 0.05 were considered statistically significant.

## Results

The study population consisted of 2552 female participants with a mean age of 44.50 (SD: 9.47) years. Baseline characteristics according to the number of parity are presented in Table 1. Generally, by increasing in the number of parity, cardiometabolic risk profiles became worse among continuous variables except for HDL-C. Hence higher parity was associated with older age, higher BMI and WC, increased BP, and higher levels of FPG, TG, and TG/HDL-C. Among categorical variables, women with one parity had a higher prevalence of current smoking but a lower prevalence of having macrosomia baby and taking antihypertensive/lipid-lowering medications. Moreover, higher educated participants were less likely to have a higher parity number.

**Table 1:** Baseline characteristics according to the number of parity: Tehran Lipid and Glucose Study

Number of parity	1	2	3	4 ≤	*P *value*
Number of participants	180	614	640	1118	
*Continuous variables, mean* ± *SD*					
Age (years)	36.46 ± 7.99	38.34 ± 6.75	42.14 ± 7.69	50.53 ± 8.07	< 0.001
BMI (kg/m^2^)	26.59 ± 4.27	27.44 ± 4.40	28.39 ± 4.43	29.59 ± 4.54	< 0.001
WC (cm)	84.48 ± 10.52	85.74 ± 10.63	88.51 ± 10.78	93.80 ± 11.15	< 0.001
SBP (mmHg)	110.66 ± 13.84	112.53 ± 13.09	116.31 ± 15.91	125.00 ± 19.93	< 0.001
DBP (mmHg)	74.44 ± 8.98	76.58 ± 9.23	78.43 ± 9.98	81.17 ± 10.82	< 0.001
FPG (mmol/L)	4.90 ± 0.50	4.92 ± 0.54	4.95 ± 0.53	5.11 ± 0.55	< 0.001
HDL-C (mmol/L)	1.18 ± 0.25	1.16 ± 0.27	1.14 ± 0.28	1.15 ± 0.30	0.316
TG (mmol/L)	1.25 (0.97)	1.41 (1.02)	1.62 (1.13)	1.84 (1.27)	< 0.001
TG/HDL-C	1.06 (1.02)	1.21 ± 1.12	1.48 ± 1.37	1.67 ± 1.49	< 0.001
*Categorical variables, n (%)*					
Smoking status					0.032
Never or former smoker	166 (92.2%)	579 (94.3%)	613 (95.8%)	1078 (96.4%)	
Current smoker	14 (7.8%)	35 (5.7%)	27 (4.2%)	40 (3.6%)	
Education level, years					< 0.001
≤ 12	146 (81.1%)	542 (88.3%)	614 (95.9%)	1097 (98.1%)	
> 12	34 (18.9%)	72 (11.7%)	26 (4.1%)	21 (1.9%)	
Low physical activity, yes	135 (75.0%)	415 (67.6%)	475 (74.2%)	833 (74.5%)	0.027
Family history of DM, yes	47 (26.1%)	174 (28.3%)	191 (29.8%)	293 (26.2%)	0.259
Antihypertensive medications, yes	4 (2.2%)	23 (3.7%)	31 (4.8%)	159 (14.2%)	< 0.001
Lipid-lowering medications, yes	3 (1.7%)	10 (1.6%)	19 (3.0%)	64 (5.7%)	< 0.001
History of macrosomia, yes	1 (0.6%)	34 (5.5%)	57 (8.9%)	141 (12.6%)	< 0.001
History of preeclampsia, yes	12 (6.7%)	51 (8.3%)	48 (7.5%)	71 (6.4%)	0.140
OCP use, yes	14 (7.8%)	60 (9.8%)	45 (7.0%)	32 (2.9%)	< 0.001

Values are shown as mean ± SD and number (%), for continuous and categorical variables, respectively; for TG values are shown as Median (interquartile range)

*SD* Standard deviation, *BMI* body mass index, *SBP* systolic blood pressure, *DBP* diastolic blood pressure, *FPG* fasting plasma glucose, *HDL-C* high density lipoprotein cholesterol, *TG* triglycerides, *DM* diabetes mellitus, *OCP* oral contraceptive pill

*The comparison *p *value between groups was calculated using ANOVA test for normal continues variables, Kruskal–Wallis test for skewed variables and chi-square test (Fisher's exact test if required) for categorical variables

From baseline phase until phase VI (2015–2018), during a median follow-up of 15.4 years (IQR: 10.7–16.4), 557 incident T2DM cases have occurred. Multivariable HRs and 95% CIs of the association of number of parity and live birth with incident T2DM are shown in Table 2 and Additional file 2: Table S1, respectively. Each additional parity and live birth was associated with 10% and 11% higher risk of incident T2DM in the age-adjusted model 1, respectively (*P* values < 0.001); the higher risk remained significant until model 3. However, after further adjustment for BMI and WC in model 4, the HR of each additional parity was attenuated significantly and reached 1.05 (95% CI 1.00–1.11; *P* value: 0.075). A similar attenuation was also occurred for the HR of each additional live birth, although it remained significant even in model 5 (1.06 [1.00–1.12; *P* value: 0.049]). Compared to women with parity/live birth number of one, those with parity/live birth number of 3 and ≥ 4 were significantly at higher risk of incident T2DM in models 1–3. After adjustment for general and central obesity indices in model 4, having ≥ 4 parity/live birth was associated with higher risk; even after further adjustment for TG/HDL-C in model 5, the risk remained marginally significant for parity ≥ 4 (1.64 [1.00–2.70; *P* value: 0.051]). Importantly, trends of the HRs across parity/live birth categories were also significant in model 1–4 and marginally significant in model 5 (*P* value: 0.055 for parity and 0.060 for live birth).

**Table 2:** Multivariable hazard ratios (HR) and 95% confidence intervals (CI) of incident T2DM by number of parity until phase VI (2015–2018): Tehran Lipid and Glucose Study

	E/N	Model 1		Model 2		Model 3		Model 4		Model 5	
		HR (95% CI)	*P* value	HR (95% CI)	*P* value	HR (95% CI)	*P* value	HR (95% CI)	*P* value	HR (95% CI)	*P* value
*Parity (continuous variable)*											
Per each additional	557/2552	1.10 (1.05–1.16)	< 0.001	1.10 (1.04–1.15)	< 0.001	1.09 (1.04–1.15)	0.001	1.05 (1.00–1.11)	0.075	1.04 (0.99–1.10)	0.108
*Number of parity*											
1	19/180	1		1		1		1		1	
2	99/614	1.48 (0.90–2.41)	0.121	1.48 (0.91–2.43)	0.116	1.48 (0.90–2.42)	0.119	1.41 (0.86–2.31)	0.172	1.39 (0.85–2.27)	0.193
3	128/640	1.74 (1.07–2.83)	0.025	1.75 (1.07–2.86)	0.025	1.73 (1.06–2.83)	0.028	1.54 (0.94–2.52)	0.084	1.50 (0.92–2.45)	0.107
≥ 4	311/1118	2.28 (1.40–3.70)	0.001	2.27 (1.39–3.72)	0.001	2.23 (1.36–3.65)	0.001	1.72 (1.05–2.83)	0.031	1.64 (1.00–2.70)	0.051
*P* value for trend			< 0.001		< 0.001		< 0.001		0.027		0.055

Model 1: Adjusted for age. Model 2: Adjusted for age, education level, low physical activity, family history of diabetes, systolic and diastolic blood pressure, and anti-hypertensive medications usage. Model 3: Model 2 + further adjusted for history of macrosomia, preeclampsia, and oral contraceptive pill (OCP) usage. Model 4: Model 3 + further adjusted for body mass index and waist circumference. Model 5: Model 4 + further adjusted for triglyceride/ high-density lipoprotein cholesterol. T2DM: type 2 diabetes mellitus; E: event; N: number

As a sensitivity analysis, to address the effect of changing BMI during follow-up on the association between parity/live birth number and incident T2DM, we rerun the Cox regression by further adjustment for BMI change during follow-up (Additional file 3: Table S2). BMI change was defined as: $\frac{{{\text{Secondary measurement}} - {\text{Baseline measurement}}}}{{{\text{Baseline measurement }} \times {\text{Time interval }}\left( {{\text{year}}} \right)}} \times 100$; secondary measurement was performed at the first follow-up after recruitment, before the individual developed T2DM or censured. The time interval was calculated as the difference of the second measurement year with the baseline recruitment. Due to the limited sample size and decrease in the number of outcomes (154 participants with incident T2DM were excluded due to the lack of secondary BMI measurement before outcome), generally, the *P* values became non-significant; however, it should be noted that even after adjustment for BMI change, the effect sizes were similar to the model 5 of the main analysis. Moreover, compared to those with parity number of one, parity ≥ 4 had a HR of 1.69 [0.94–3.01; *P* value: 0.078], and the trend of the HRs across parity categories was marginally significant (*P* value: 0.064).

As another sensitivity analysis, we reexamined the impact of parity/live birth on incident T2DM during follow-up of less than 10 years. Accordingly, during a median follow-up of 9.4 years (IQR: 8.6–10.4), 323 incident T2DM cases have occurred until phase IV (2008–2011). In model 1–3, each additional parity and live birth caused a higher risk of incident T2DM, and also those with parity/live birth number of ≥ 4 were at higher risk; however, after adjustment for obesity indices in model 4, the higher risk became non-significant (Table 3 and Additional file 4: Table S3).

**Table 3:** Multivariable hazard ratios (HR) and 95% confidence intervals (CI) of incident T2DM by number of parity until phase IV (2008–2011): Tehran Lipid and Glucose Study

	E/N	Model 1		Model 2		Model 3		Model 4		Model 5	
		HR (95% CI)	*P* value	HR (95% CI)	*P* value	HR (95% CI)	*P* value	HR (95% CI)	*P* value	HR (95% CI)	*P* value
*Parity (continuous variable)*											
Per each additional	323/2492	1.12 (1.04–1.19)	0.001	1.11 (1.04–1.19)	0.003	1.10 (1.03–1.18)	0.007	1.06 (0.99–1.13)	0.118	1.06 (0.98–1.13)	0.126
*Number of parity*											
1	10/173	1		1		1		1		1	
2	54/597	1.51 (0.77–2.97)	0.229	1.51 (0.77–2.97)	0.234	1.49 (0.76–2.93)	0.249	1.42 (0.72–2.80)	0.307	1.40 (0.71–2.75)	0.335
3	72/624	1.74 (0.89–3.38)	0.104	1.72 (0.88–3.36)	0.114	1.67 (0.85–3.28)	0.134	1.50 (0.77–2.95)	0.235	1.45 (0.74–2.85)	0.276
≥ 4	187/1098	2.16 (1.11–4.20)	0.023	2.11 (1.08–4.13)	0.029	2.03 (1.03–3.98)	0.040	1.55 (0.79–3.05)	0.200	1.47 (0.75–2.89)	0.267
*P *value for trend			0.006		0.010		0.017		0.298		0.432

Model 1: Adjusted for age. Model 2: Adjusted for age, education level, low physical activity, family history of diabetes, systolic and diastolic blood pressure, and anti-hypertensive medications usage. Model 3: Model 2 + further adjusted for history of macrosomia, preeclampsia, and oral contraceptive pill (OCP) usage. Model 4: Model 3 + further adjusted for body mass index and waist circumference. Model 5: Model 4 + further adjusted for triglyceride/ high-density lipoprotein cholesterol. T2DM: type 2 diabetes mellitus; E: event; N: number

## Discussion

This is the first population-based cohort study conducted in the MENA region that examined the effect of the parity/live birth number on incident T2DM. During 15.4 years of follow-up, after adjustment for a wide series of important T2DM risk factors, including age, education level, family history of diabetes, SBP, DBP, using anti-hypertensive medications, and reproductive factors, a higher parity/live birth number was associated with higher risk of T2DM development. After further adjustment for obesity indices and TG/HDL-C, the higher risk was attenuated; however, the trends of the HRs across parity/live birth categories were marginally significant, and those with parity ≥ 4 had more than 60% higher risk for T2DM development. Moreover, each additional live birth was associated with 6% significant higher risk for incident T2DM.

Our findings were in line with two previous meta-analysis studies [5, 9]. Li et al., in a meta-analysis of the cohort, cross-sectional, and case–control studies found a nonlinear association between parity and the risk of T2DM in which the combined multivariable RR for T2DM development was 1.06 (95% CI: 1.02–1.09) per live birth, with high heterogeneity (I^2^ = 87.2%) [5]. Also, in their sensitivity analysis for prospective studies only, the RR reached 1.09 [5]. Similarly, in another mate-analysis of cohort studies by Guo et al., each unit increase in parity number had the combined RR of 1.06 (95% CI 1.02–1.09); however, a significant high heterogeneity was shown between included studies (I^2^ = 84.3%) [9]. Moreover, in both meta-analyses, compared to those with lower parity (reference = 0 or 1), participants of prospective studies who had higher parity (at least 4 live births) showed about 40% higher risk for T2DM development [5, 9]. Based on data of 126,721 middle-aged women from eight cohort studies, a U-shaped association was found between the number of children and the risk of DM, in which women with 2 live births had the lowest risk [16]. A similar U-shaped relation was also observed among Danish women older than 33 years of age [17] and Canadian women aged 18–50 years [10].

In the coronary artery risk development in young adults (CARDIA) Study, it was shown that childbearing did not increase the incidence of diabetes as long as women never developed gestational diabetes mellitus (GDM). In other words, the association of childbearing with incident DM can be explained through GDM [18]. On the other hand, among Canadian women, an increased risk of DM with increasing parity was observed, even after adjustment for GDM [10]. Similarly, among Danish women without a history of GDM, a relationship between parity and incident DM was also found [17]. In the current study, even after adjustment for macrosomia, as a surrogate of GDM [19], the association of parity/live birth number and incident T2DM remained significant.

During a 5-year follow-up of Japanese women, Nanri et al. found that the association of parity with T2DM development risk was significantly attenuated after adjustment for BMI. They suggested that a higher risk of T2DM development due to increased parity might be partly explained by weight retention after pregnancy [20]. In our short-term follow-up of less than 10 years, we also found similar results that all *P* values became non-significant after adjustment for obesity indices in model 4. However, in our long-term follow-up (main analysis), similar to a cohort study among American women [21], although the effect sizes for higher parity/live birth numbers were attenuated prominently in model 4, but remained significant for parity/live birth number of ≥ 4. Furthermore, when we considered BMI change during follow-up as another covariate, generally, the effect sizes did not change, and the *P* value for the trend of HRs across categories of parity number remained marginally significant.

In addition to weight retention, another explanation for the association of the parity/live birth number with incident T2DM in women can be the insulin resistance (IR) pathway. During a normal pregnancy, the target tissues (liver, muscle, or adipose tissue) of the mother become increasingly insensitive to insulin, mainly due to hormone production by the placenta that antagonizes insulin [22]. This IR may progress to GDM or might be overcome by a sufficient increase in insulin production by pancreatic beta cells [22]; however, this process can lead to an extra burden on β-cell function and affect insulin secretion, even after delivery [9, 23]. In addition to GDM, a well-established risk factor for developing T2DM in the future [24], it is also known that mothers with milder degrees of dysglycemia during pregnancy (i.e., less severe than GDM) were at higher risk for developing prediabetes and T2DM in the future [23, 25]. Moreover, in full-multivariable analysis, Kramer et al., found that insulin sensitivity (as assessed by Matsuda index) decreased during the first 3 years postpartum, even among women who had had a normal glucose-tolerant pregnancy [23]. Moreover, the expected reduction in the physical activity and some cardiometabolic changes during pregnancy (weight gain and dyslipidemia) can exacerbate this IR pathway [22]. By repeating pregnancy, exposure time to IR condition increases and can lead to a higher chance of T2DM development. In the current study, despite the lack of data on insulin measurement, after further adjustment for TG/HDL-C (surrogate of IR [26, 27]), each additional live birth and parity ≥ 4 were generally associated with higher risk. It means that other factors might also have roles in the association of childbearing and T2DM development, especially psycho-socioeconomic factors.

The present study has several strengths, including standardized measurements of traditional risk factors and using oral glucose tolerance test for outcome definition, rather than relying on self-reported data. Moreover, besides the well-known risk factors of T2DM, we also considered BMI change as a strong covariate in our analysis, which was not addressed in previous studies of this field. The current study also has several weaknesses. First, during follow-up, possible changes in risk factors, as well as the parity/live birth number, were not considered. Second, we were not able to show the impact of nulliparity due to the limited number of participants with no live birth. Third, we did not have access to data on participant diet, job status, income, and psychiatric history, which are the potential representative factors of the socioeconomic and lifestyle status. They might affect the association between the parity/live birth number and incident T2DM. Fourth, at the baseline of the current study, only 18 participants reported a positive history of GDM that was too low to be considered as a covariate. Moreover, at the time of recruitment of this study (1999–2001) and before, there was no routine screening program for GDM like oral glucose tolerance test (OGTT) during prenatal care in Iran. Therefore, most of the GDM cases were missed at that time. However, in the current study, we adjusted our models for macrosomia, the main surrogate of GDM. Finally, this study was conducted among residents of Tehran city, and its findings may not be extendable to rural populations.

## Conclusion

In this population-based observational cohort study, during a long-term follow-up, after adjustment for potential T2DM risk factors, reproductive factors, obesity indices, and TG/HDL-C (IR surrogate), we demonstrated that higher parity/live birth numbers could be associated with increased risk of T2DM development. Moreover, even after further adjustment for BMI change, the suggestive higher risk was still found. We suggested that weight retention can have a key role between parity/live birth number and incident T2DM, and weight management should be more considered after delivery. Moreover, we recommended that the potential role of psycho-socioeconomic factors should be investigated in future studies.

## Supplementary Information

**Additional file 1**. Tehran Lipid and Glucose Prospective Study General Questionnaire.

**Additional file 2. Table S1.** Multivariable hazard ratios (HR) and 95% confidence intervals (CI) of incident T2DM by number of live birth until phase VI (2015-2018): Tehran Lipid and Glucose Study.

**Additional file 3. Table S2.** Multivariable hazard ratios (HR) and 95% confidence intervals (CI) of incident T2DM by parity/live birth number, further adjusted for BMI change during follow-up: Tehran Lipid and Glucose Study.

**Additional file 4. Table S3.** Multivariable hazard ratios (HR) and 95% confidence intervals (CI) of incident T2DM by number of live birth until phase IV (2008-2011): Tehran Lipid and Glucose Study.

## Data Availability

The datasets used and/or analysed during the current study are available from the corresponding author on reasonable request.

## Abbreviations

DM: Diabetes mellitus
DALYs: Disability-adjusted life-years
T2DM: Type 2 diabetes mellitus
RR: Relative risk
CI: Confidence interval
MENA: Middle East and North Africa
TLGS: Tehran Lipid and Glucose Study
NCD: Non-communicable disease
FPG: Fasting plasma glucose
2h-PCPG: 2-Hour post-challenge plasma glucose
LRC: Lipid Research Clinic
WC: Waist circumference
BMI: Body mass index
SBP: Systolic blood pressure
DBP: Diastolic blood pressure
HDL-C: High-density lipoprotein cholesterol
TG: Triglycerides
SD: Standard deviation
IQR: Interquartile range
HR: Hazard ratio
OCP: Oral contraceptive pill
CARDIA: Coronary artery risk development in young adults
GDM: Gestational diabetes mellitus
IR: Insulin resistance
OGTT: Oral glucose tolerance test
