# Supplementary material for: The association of parity/live birth number with incident type 2 diabetes among women: over 15 years of follow-up in The Tehran Lipid and Glucose Study
Source: BMC Womens Health. 2021 Oct 29;21:378. doi: 10.1186/s12905-021-01519-7 (PMC8556972; doi:10.1186/s12905-021-01519-7)
Supplement: Supplementary file 1 — Additional file 1. Tehran Lipid and Glucose Prospective Study General Questionnaire. [file 12905_2021_1519_MOESM1_ESM.pdf]

In the Name of God
Shaheed Beheshti University of Medical Sciences

Endocrine Research Center

Tehran Lipid and Glucose Prospective Study General Questionnaire

Note: Each question is specific to a special sex and age group.

A) Questions on demographic characteristics:

1- Family name: .... **2- Name:** .... **3- Father's name:** ....

4- Address: ....

5- Where do you work?(address) ....

....

**6- Close relative Tel:**	☐☐☐☐☐☐☐
**7- Date of interview:**	☐☐ ☐☐ ☐☐
**8- Admission No:**	☐☐☐☐☐
**9- Household No:**	☐☐☐☐☐
**10- Cluster No:**	☐☐☐☐☐
**11- Identification No:**	☐☐☐☐☐☐☐☐☐☐
**12- Birth Date according to Identification document:dd/mm/yy**	☐☐ ☐☐ ☐☐

A) Questions on demographic characteristics:	
**13- Actual birth date: dd/mm/yy**	☐☐ ☐☐ ☐☐
**14- Age:**	☐☐
**15- Sex** 1- Male 2- Female	☐
**16- Marital status** 1- Single 2- Married 3- Divorced 4- Widowed	☐
**17- Literacy (>6 years old)** 1- Illiterate 2- Literate	☐
**18- Are you engaged in educational programs now (>6 years old)?** 1-Yes 2- No	☐
**19- What is your educational Level (>6 years old)?** 1- Primary School 2- 6-8 th grader 3- 9-11 th grader 4- Technician 5- Bachelor 6- Master 7- GP 8- Specialist 9- Sub specialist 10- Nehzat 11- Religious Sciences	☐☐

A) Questions on demographic characteristics:	
**20- What is your highest educational certificate (go to question 21)?**<br>1- Primary School 7- Master<br>2- 6-8th grade 8- General practitioner<br>3- 9-11th grade 9- Specialist<br>4- High school 10- Nehzat<br>5- Technician 11- Religious Sciences<br>6- Bachelor	☐☐
**21- What has your occupational status been during the past month (>10 years old) ?**<br>1- Employed 4- Housewife<br>2- Unemployed 5- Unemployed, having income<br>3- Student 6- Others<br>Note: If you choose any of No. 2,3,4,5,6 go to question 24.	☐
**22- What is your job position (>10 years old)?**	....
**23- What is your job group?**	....
**24- How many years have you been residing in Tehran (as a permanent resident)?**	....

B) Questions about past medical history:

**1- Have you ever been informed about following disorders by a physician?**<br>1- Yes 2- No 9- Don't know<br>1-1 Hyperlipidemia<br>1-2 Hyperglycemia<br>1-3 Hypertension<br>1-4 Ischemic heart disease<br>Please explain:<br>1-5 Non-Ischemic heart disease<br>Please explain:<br>1-6 Have you had past history of chronic diarrohea?<br>1-7 Have you experienced significant weight loss during previous 6 months?<br>1-8 Stroke<br>1-9 Malignant Disorders<br>1-10 Have you ever had a major and important medical problem?<br>Address it:	☐ -☐☐
**2- Have you ever been hospitalized so far?**<br>1-Yes: Please explain:<br>2- No(go to question 4)	☐
**3-Have you been hospitalized during the last 3 months?**<br>1- Yes 2- No	☐
**4- Is there any history of MI, stroke or sudden death, in your mother's/ sister's/ daughter's past medical history, under the age of 65?**<br>1- Yes 2- No 3- Don't know	☐

**B) Questions about past medical history:**	
**5- Is there any history of MI, stroke or sudden death, in your father's/ brother's/ son's past medical history, under the age of 55?**<br>1- Yes 2- No 3- Don't know	☐
**6- Has any of your first-degree relatives (father, mother, brother, sister, son, daughter) had diabetes mellitus?**<br>1- Yes 2- No 3- Don't know	☐
**7- Have you ever taken any of following medications during the last month (Oral or injection)?**<br>1-Yes regularly 2- No<br>3- Yes, irregularly 4- Don't know<br><br>7-1) Antidiabetic drugs<br>7-2) Antihyperlipidemic drugs<br>7-3) Diuretics<br>7-4) β- blockers<br>7-5) Antihypertensive drugs<br>7-6) Corticosteroids<br>7-7) Female hormones<br>7-8) Male hormones<br>7-9) Thyroid drugs<br>7-10) Antithyroid drugs<br>7-11) Aspirin (A.S.A)	☐☐

C) Questions on personal habits:

**1-Do you smoke cigarettes?**<br>1- Yes, regularly 2- No (go to question 3)<br>3- Yes, irregularly (go to question 3)	☐
**2-On the average , how many cigarettes do you smoke per day ?**<br>1) 1-5 2) 6-10 3) 11-15 4) 16-20 5) >20<br>6) insufficient data<br>Then go to question 5	☐
**3- Have you ever smoked cigarettes in the past?**<br>1- Yes, regularly 2- No (go to question 6)<br>3- Yes, irregularly (go to question 6)	☐
**4- How many years have you stopped smoking?**<br>(99- Don't know)	☐☐
**5- How old were you when you first started to smoke regularly?** (99- Don't know)	☐☐
**6- Do you smoke pipe now?**<br>1- Yes, regularly 2- No (go to question 9)<br>3- Yes, irregularly	☐
**7- How many days does a pack of pipe tobacco last?**<br>(99- Don't know)	☐☐

C) Questions on personal habis:	
**8- How old were you when you first started to smoke pipe?**<br>(99- Don't know)	☐☐
**9- Have you ever smoked a pipe in the past?**<br>1- Yes, regularly 2- No<br>3- Yes, irregularly	☐
**10- Do you smoke hookah (ghalian) ?**<br>1- Yes, regularly 2- No (go to question 13)<br>3- Yes, irregularly	☐
**11- How many days does a pack of tobacco last for hookah (ghalian) ?** (99- Don't know)	☐☐
**12- How old were you when you first started to smoke hookah (ghalian) ?** (99- Don't know)<br>Then go to question 14.	☐☐
**13- Have you ever smoked hookah (ghalian) in the past?**<br>1- Yes, regularly 2- No 3- Yes, irregularly	☐

The following questions are asked from all age groups:

**14- Is there any cigarette smoker in your house that you inhale his/ her tobacco smoke?**<br>1- Yes 2- No	☐

C) Questions on personal habits:	
**15- Is there any cigarette smoker at your work that you inhale his/ her tobacco smoke?**<br>1- Yes 2- No<br>If both of your answers are no, then go to question 20.	☐
**16- How many smokers are there totally, in the house or at work, that you are closely subjected to their smoke?**	☐
**17- On the average, how many days a week, are you closely subjected to their tobacco smoke?**<br>1) 1-2 days 2) 3-5 days 3) 6-7 days 9) Don't know	☐
**18- On the average, how many hours a day are you closely subjected to their tobacco smoke?**<br>1) <1 hour 2) 1-3 hours 3) >3 hours 9) Don't know	☐
**19- On the average, how many cigarettes do they smoke each day?**<br>1) 1-10 2) 11-20 3) >20 9) Don't know	☐

C) Questions on personal habits:

The following questions are asked from persons >15 years old

**20- Compared to other people in your age and sex, how would you rate your physical activity at work?**<br>1- More active 2- About the same<br>3- Less active 4- Not applicable	☐
**21- Compared to other people in your age and sex how would you rate your physical activity out of work?**<br>1- More active 2- About the same<br>3- Less active 4- Not applicable	☐
**22- Do you regularly engage in strenuous activities or hard physical labour?**<br>1- Yes 2- No	☐
**23- Do you exercise or labour at least three times a week?**<br>1- Yes 2- No	☐

D) Questions on chest pain and claudication:

**1- Have you ever had any pain or discomfort in your chest?**<br>1- Yes 2- No<br>If "no" proceed to section C.<br>If "Yes" ask next question.	☐
**2- Do you get it when you walk uphill or hurry?**<br>1- Yes 2- No<br>3-Never hurries or walks uphill	☐
**3- Do you get it when you walk at an ordinary pace on the level ground?**<br>1- Yes 2- No	☐
**4- What do you do if you get it while you are walking?**<br>1-Stop or slow down 2- Carry onrecord "stop or slow down" if subject carries on after taking nitroglycerine.	☐
**5- If you stand still, what happens to the pain?**<br>1- It relieves 2-It doesn't relieve	☐
**6- How soon?**<br>1- 10 minutes or less 2- More than 10 minutes	☐

**D) Questions on chest pain and claudication:**	
**7- Can you show me where the pain was?**<br>1- Sternum (upper or middle) 2- Sternum (lower)<br>3- Left anterior chest 4- Left arm<br>5- other<br>Record all areas mentioned.	…………………………
**8- Do you feel it anywhere else?**<br>1-Yes 2- No<br>If yes record additional information above.	☐
**Section B: Possible infarction**	
**9- Have you ever had a severe pain across the front of your chest lasting for half an hour or more?**<br>1-Yes 2- No	☐
**Section C: Intermittent claudication**<br>***If an answer is recorded in a box no further questions need to be asked.***	
**10- Do you get pain in either leg on walking?**<br>1-Yes 2- No	☐

D) Questions on chest pain and claudication:<br>***Section C:***	
**11- Does this pain ever begin when you are standing still or sitting?**<br>1-Yes 2- No	☐
**12-In what part of your leg do you feel it?**<br>Pain includes calf/ calves<br>Pain does not include calf/ calves	☐
**13- Do you get it if you walk uphill or hurry?**<br>1- Yes 2-No 3-Never hurries or walks uphill	☐
**14- Do you get it if you walk at an ordinary pace on the ground level?**<br>1- Yes 2-No	☐
**15- Does the pain ever disappears while you are walking?**<br>1- Yes 2-No	☐
**16- What do you do if you get it while you are walking?**<br>1- Stop or slow down 2- Carry on	☐
**17-What happens to it if you stand still?**<br>1- Relieved 2- Not relieved	☐
**18- How soon?**<br>1-10 minutes or less 2- More than 10 minutes	☐

E) The following questions are asked from women only:	
**1-Do you have normal menstrual cycles? (for cases>10years of age)**<br>1-Yes, naturally without taking control pills(go to question 5)<br>2- No (go to question 13)<br>3- Prepubertal age (go to physical exam section)<br>4- Yes, but with hormone replacement pills	☐
**2-Are you currently taking any hormone replacement medication?**<br>1- Estrogen 2- Progesterone 3-Both 4-None	☐
**3- For how long has your menstrual cycles stopped? (Cases >15 years of ages) (Month and year)**	***Year*** ☐☐<br>***Month*** ☐☐
**4- What is the reason of your menstrual cycles stopping?**<br>1- Natural menopause<br>2- Early-onset menopause<br>3- Hysterectomy without oophorectomy or unilateral oophorectomy<br>4- Hysterectomy + bilateral oophorectomy<br>5- Bilateral oophorectomy without hysterectomy<br>6- Other<br>7- Don’t know	☐

E) The following questions are for married women only:	
**5- Which contraception method have you used during the last three months?**<br>1-LD contraceptive 2-HD contraceptive<br>3-Minipill 4- Triphasic pill	☐
**6- Are you pregnant now?**<br>1-Yes 2-No 3-Don't know	☐
**7- Have you ever been pregnant?**<br>1- Yes 2-No (go to physical exam section)	☐
**8- How many live born children have you had?**	☐☐
**9- Have you given birth to a baby in the past one year?**<br>1- Yes 2-No	☐
**10- Have you ever had any abortion?**<br>1- Yes 2- No (go to question 14)	☐
**11- How many abortions have you had?**	☐
**12- Do you have any history of stillbirth?**<br>1- Yes 2- No (go to question 14)	☐

F) The following questions are for married women only:	
**13- How many stillbirths have you ever experienced?**	☐☐
**14- Has a doctor ever told you that you had hypertension or convulsion during any of your pregnancies?**<br>1- Yes 2- No 3- Unknown	☐
**15- Has a doctor ever told you that you had high blood glucose or gestational diabetes during any of your pregnancies?**<br>1-Yes 2- No 3- Unknown	☐
**16- Have you ever had a baby that weighed 4.5 Kg or more?**<br>1-Yes 2- No 3- Unknown	☐
**17- Are you breast-feeding now?**<br>1-Yes 2- No 3- Unknown	☐

G) Physical Examination Summery:	
**1- Pulse per minute:**	☐☐
**2-Sphygmomanometer's cuff size:**<br>1-Small 2-Medium 3-Large	☐
**3- Systolic blood pressure (mmHg), first measurement:**	☐☐☐
**4-Diastolic blood pressure (mmHg), first measurement:**	☐☐☐
**5- Systolic blood pressure (mmHg), second measurement:**	☐☐☐
**6- Diastolic blood pressure (mmHg), second measurement:**	☐☐☐
**7- The time of first blood pressure measurement:**	☐☐ ☐☐
**8- Room temperature (°C)**	☐☐
**9- Goiter**<br>1- Yes 2- No	☐

G) Physical Examination Summery:	
**10- Goiter grading**<br>1- Grade I 2- Grade II	☐
**11- Thyroid nodule**<br>1- Yes 2- No	☐
**12-Anthropometry**<br>1- Height (cm)<br>2- Weight (kg)<br>3- Waist circumference (cm)<br>4- Hip circumference (cm)<br>5- Wrist circumference (cm with one decimal digit)	……………………<br>……………………<br>……………………<br>……………………<br>……………………
