# Supplementary material for: The association of parity/live birth number with incident type 2 diabetes among women: over 15 years of follow-up in The Tehran Lipid and Glucose Study
Source: BMC Womens Health. 2021 Oct 29;21:378. doi: 10.1186/s12905-021-01519-7 (PMC8556972; doi:10.1186/s12905-021-01519-7)
Supplement: Supplementary file 2 — Additional file 2. Table S1. Multivariable hazard ratios (HR) and 95% confidence intervals (CI) of incident T2DM by number of live birth until phase VI (2015-2018): Tehran Lipid and Glucose Study. [file 12905_2021_1519_MOESM2_ESM.docx]

| **Table S1. Multivariable hazard ratios (HR) and 95% confidence intervals (CI) of incident T2DM by number of live birth until phase Ⅵ (2015-2018): Tehran Lipid and Glucose Study.** | | | | | | | | | | | |
| --- | --- | --- | --- | --- | --- | --- | --- | --- | --- | --- | --- |
|  | **E/N** | **Model 1** | | **Model 2** | | **Model 3** | | **Model 4** | | **Model 5** | |
|  |  | **HR**  **(95% CI)** | **P-value** | **HR**  **(95% CI)** | **P-value** | **HR**  **(95% CI)** | **P-value** | **HR**  **(95% CI)** | **P-value** | **HR**  **(95% CI)** | **P-value** |
|  |  |  |  |  |  |  |  |  |  |  |  |
| **Live birth (continuous variable)** |  |  |  |  |  |  |  |  |  |  |  |
| - Per each additional | 557/2,552 | 1.11  (1.05-1.17) | < 0.001 | 1.11  (1.05-1.17) | < 0.001 | 1.11  (1.05-1.17) | < 0.001 | 1.06  (1.00-1.12) | 0.039 | 1.06  (1.00-1.12) | 0.049 |
| **Number of Live birth** |  |  |  |  |  |  |  |  |  |  |  |
| - 1 | 22/191 | 1 |  | 1 |  | 1 |  | 1 |  | 1 |  |
| - 2 | 102/643 | 1.33  (0.84-2.11) | 0.224 | 1.36  (0.86-2.17) | 0.189 | 1.36  (0.86-2.16) | 0.194 | 1.28  (0.80-2.03) | 0.303 | 1.26  (0.79-2.00) | 0.325 |
| - 3 | 134/648 | 1.66  (1.05-2.62) | 0.029 | 1.69  (1.06-2.68) | 0.026 | 1.67  (1.05-2.65) | 0.029 | 1.48  (0.93-2.35) | 0.096 | 1.45  (0.91-2.30) | 0.117 |
| - ≥ 4 | 299/1,070 | 2.10  (1.33-3.31) | 0.002 | 2.13  (1.34-3.39) | 0.001 | 2.08  (1.31-3.32) | 0.002 | 1.60  (1.00-2.55) | 0.050 | 1.52  (0.95-2.43) | 0.079 |
| - P-value for trend |  |  | < 0.001 |  | < 0.001 |  | < 0.001 |  | 0.029 |  | 0.060 |
| Model 1: Adjusted for age.  Model 2: Adjusted for age, education level, low physical activity, family history of diabetes, systolic and diastolic blood pressure, and anti-hypertensive medications usage.  Model 3: Model 2 + further adjusted for history of macrosomia, preeclampsia, and oral contraceptive pill (OCP) usage.  Model 4: Model 3 + further adjusted for body mass index and waist circumference.  Model 5: Model 4 + further adjusted for triglyceride/ high-density lipoprotein cholesterol.  T2DM: type 2 diabetes mellitus; E: event; N: number. | | | | | | | | | | | |
