# Supplementary material for: The association of parity/live birth number with incident type 2 diabetes among women: over 15 years of follow-up in The Tehran Lipid and Glucose Study
Source: BMC Womens Health. 2021 Oct 29;21:378. doi: 10.1186/s12905-021-01519-7 (PMC8556972; doi:10.1186/s12905-021-01519-7)
Supplement: Supplementary file 3 — Additional file 3. Table S2. Multivariable hazard ratios (HR) and 95% confidence intervals (CI) of incident T2DM by parity/live birth number, further adjusted for BMI change during follow-up: Tehran Lipid and Glucose Study. [file 12905_2021_1519_MOESM3_ESM.docx]

| **Table S2. Multivariable hazard ratios (HR) and 95% confidence intervals (CI) of incident T2DM by parity/live birth number, further adjusted for BMI change during follow-up: Tehran Lipid and Glucose Study.** | | | | | |
| --- | --- | --- | --- | --- | --- |
|  | **HR (95% CI)** | **P-value** |  | **HR (95% CI)** | **P-value** |
| **Parity (continuous variable)** |  |  | **Live birth (continuous variable)** |  |  |
| - Per each additional | 1.05 (0.98-1.12) | 0.150 | - Per each additional | 1.05 (0.98-1.13) | 0.153 |
| **Number of Parity** |  |  | **Number of Live birth** |  |  |
| - 1 | 1 |  | - 1 | 1 |  |
| - 2 | 1.38 (0.78-2.45) | 0.272 | - 2 | 1.30 (0.76-2.23) | 0.342 |
| - 3 | 1.45 (0.82-2.58) | 0.205 | - 3 | 1.41 (0.82-2.43) | 0.215 |
| - ≥ 4 | 1.69 (0.94-3.01) | 0.078 | - ≥ 4 | 1.53 (0.88-2.65) | 0.129 |
| - P-value for trend |  | 0.064 | - P-value for trend |  | 0.125 |
| Adjusted for age, education level, low physical activity, family history of diabetes, systolic and diastolic blood pressure, anti-hypertensive medications usage, history of macrosomia, preeclampsia, oral contraceptive pill (OCP) usage, triglyceride/ high-density lipoprotein cholesterol (TG/HDL-C), body mass index (BMI), waist circumference, and BMI change during follow-up. | | | | | |
