# Supplementary material for: The association of parity/live birth number with incident type 2 diabetes among women: over 15 years of follow-up in The Tehran Lipid and Glucose Study
Source: BMC Womens Health. 2021 Oct 29;21:378. doi: 10.1186/s12905-021-01519-7 (PMC8556972; doi:10.1186/s12905-021-01519-7)
Supplement: Supplementary file 4 — Additional file 4. Table S3. Multivariable hazard ratios (HR) and 95% confidence intervals (CI) of incident T2DM by number of live birth until phase IV (2008-2011): Tehran Lipid and Glucose Study. [file 12905_2021_1519_MOESM4_ESM.docx]

| **Table S3. Multivariable hazard ratios (HR) and 95% confidence intervals (CI) of incident T2DM by number of live birth until phase Ⅳ (2008-2011): Tehran Lipid and Glucose Study.** | | | | | | | | | | | |
| --- | --- | --- | --- | --- | --- | --- | --- | --- | --- | --- | --- |
|  | **E/N** | **Model 1** | | **Model 2** | | **Model 3** | | **Model 4** | | **Model 5** | |
|  |  | **HR**  **(95% CI)** | **P-value** | **HR**  **(95% CI)** | **P-value** | **HR**  **(95% CI)** | **P-value** | **HR**  **(95% CI)** | **P-value** | **HR**  **(95% CI)** | **P-value** |
|  |  |  |  |  |  |  |  |  |  |  |  |
| **Live birth (continuous variable)** |  |  |  |  |  |  |  |  |  |  |  |
| - Per each additional | 323/2,492 | 1.12  (1.05-1.20) | 0.001 | 1.11  (1.04-1.20) | 0.002 | 1.11  (1.03-1.19) | 0.006 | 1.06  (0.99-1.14) | 0.109 | 1.06  (0.98-1.14) | 0.123 |
| **Number of Live birth** |  |  |  |  |  |  |  |  |  |  |  |
| - 1 | 12/184 | 1 |  | 1 |  | 1 |  | 1 |  | 1 |  |
| - 2 | 56/626 | 1.33  (0.71-2.48) | 0.372 | 1.35  (0.72-2.53) | 0.334 | 1.33  (0.71-2.50) | 0.367 | 1.26  (0.67-2.35) | 0.473 | 1.24  (0.66-2.32) | 0.500 |
| - 3 | 73/632 | 1.55  (0.84-2.87) | 0.164 | 1.55  (0.83-2.89) | 0.166 | 1.51  (0.81-2.82) | 0.195 | 1.35  (0.72-2.52) | 0.345 | 1.32  (0.71-2.46) | 0.384 |
| - ≥ 4 | 182/1,050 | 1.98  (1.07-3.65) | 0.030 | 1.98  (1.06-3.68) | 0.031 | 1.89  (1.01-3.53) | 0.046 | 1.45  (0.78-2.71) | 0.244 | 1.37  (0.73-2.57) | 0.320 |
| - P-value for trend |  |  | 0.004 |  | 0.007 |  | 0.013 |  | 0.234 |  | 0.351 |
| Model 1: Adjusted for age.  Model 2: Adjusted for age, education level, low physical activity, family history of diabetes, systolic and diastolic blood pressure, and anti-hypertensive medications usage.  Model 3: Model 2 + further adjusted for history of macrosomia, preeclampsia, and oral contraceptive pill (OCP) usage.  Model 4: Model 3 + further adjusted for body mass index and waist circumference.  Model 5: Model 4 + further adjusted for triglyceride/ high-density lipoprotein cholesterol.  T2DM: type 2 diabetes mellitus; E: event; N: number. | | | | | | | | | | | |
